# Compartmentalized immune responses and the local microbiota determine mucosal and systemic immunity against SARS-CoV-2

**DOI:** 10.1038/s41423-021-00822-5

**Published:** 2022-01-07

**Authors:** Jonas Buttenschön, Stefan Vogt, Jochen Mattner

**Affiliations:** 1 Mikrobiologisches Institut – Klinische Mikrobiologie, Immunologie und Hygiene, Universitätsklinikum Erlangen and Friedrich-Alexander-Universität (FAU) Erlangen-Nürnberg, Erlangen, Germany; 2 Medical Immunology Campus Erlangen, FAU Erlangen-Nürnberg, Erlangen, Germany

**Keywords:** Viral infection, Predictive markers

The clinical manifestations of SARS-CoV-2 infection, which is the cause of the coronavirus disease 2019 (COVID-19) pandemic, are highly variable and range from asymptomatic carriage or mild symptoms to severe disease involving different organ systems. However, the specific factors influencing individual clinical outcomes remain unclear. Thus, to characterize the versatile interplay of mucosal and systemic immune responses with the local microbiome and the viral load, as well as its impact on the course of the disease, Smith et al. performed integrated analyses of nasopharyngeal swabs and plasma samples from COVID-19 patients with varying degrees of illness severity. They observed that spike-specific neutralizing antibodies were heterogeneous between paired plasma samples and nasopharyngeal swabs from individual SARS-CoV-2 patients, suggesting a tissue-dependent regulation of humoral immune responses. Their study also confirmed that systemic inflammatory responses were associated with viral load. Interestingly, dysbiosis of the local microbiota and an accumulation of (facultative) pathogenic bacteria in the nasopharynx, frequently reported in secondary respiratory infections, were linked to mucosal inflammation and severe COVID-19. Conversely, the levels of cytokines, including IL-33 and different interferons that might be important for viral control, were reduced. Thus, Smith et al. revealed the nasopharyngeal microbiome as a novel player influencing local and systemic immune responses to SARS-CoV-2 and subsequently the clinical outcome of COVID-19 [1].

The clinical manifestations following SARS-CoV-2 infection are highly variable. Viral replication, as well as comorbidities, age, sex, the immune response of the host, or a combination of any or all of these factors, have been suggested to influence the outcome of disease [2, 3]. Although it is known that SARS-CoV-2 suppresses innate and antiviral immunity, causes peripheral lymphopenia, and promotes hyperinflammatory macrophage/monocyte activation [4], the distinct pathophysiologic mechanisms underlying severe COVID-19 have remained largely unknown. Thus, to characterize disease severity-determining factors, Smith et al. studied 15 patients with moderate, 11 with severe, and 23 with critical COVID-19 at the time of hospitalization as well as 12 age- and sex-matched healthy controls [1]. SARS-CoV-2 infections were confirmed or excluded by PCR. Plasma and nasopharyngeal swab samples were collected from patients and controls 8–12 days after the onset of disease, and systemic as well as mucosal SARS-CoV-2 spike-specific antibodies, cytokines, viral loads, and bacterial communities were evaluated.

The authors described, dependent on the severity of disease, an increasing frequency and intensity of spike-specific IgG and IgA antibodies in plasma and nasopharyngeal samples of patients, as reported in previous studies [5, 6]. The neutralization activity of these antibodies also increased with the severity of the disease. Spike-specific IgG or IgA antibodies, however, were substantially less frequent in nasopharyngeal secretions than in the blood. Surprisingly, there was only a poor correlation of the antibody titers and their neutralization capacity between plasma and nasopharyngeal samples within a single individual. Interestingly, two patients with moderate disease developed antibodies neither in their plasma nor in their nasopharyngeal secretion, while two other patients—with the critical disease—did not seroconvert but exhibited strong nasopharyngeal antibody responses. Thus, a tissue site-specific regulation of the humoral immune response in SARS-CoV-2 patients exists at the early stages of the disease. Indeed, the clinical outcome of infection in these four patients compared to the rest of the COVID-19 cohort will be of interest.

Of the 46 cytokines quantified in this study, 13 and 7 cytokines showed different levels between plasma and nasopharyngeal samples of healthy controls and patients with COVID-19, respectively. These differences included enhanced concentrations of different inflammatory cytokines in the plasma and decreased concentrations of interferons in mucus secretions, as previously reported [7, 8]. Smith et al. confirmed that interindividual differences in total protein or mucus content did not account for the differences observed in nasopharyngeal cytokine concentrations. Importantly, the concentrations of IL-6, IL-10, fibroblast growth factor, PD-L1, TNF, IL-1β, and IL-1RA were significantly increased in the plasma of patients with severe disease, while that of IFN-α2 was decreased. In contrast, fms-related receptor tyrosine kinase 3 ligand, epidermal growth factor (EGF), CXCL1 (GROα), PDGF-AA, IL-7, and TGF-α accumulated in significantly greater amounts in nasopharyngeal samples with worsening disease. Only CCL2 and VEGF levels were enhanced in tissue samples from both tissue sites. Thus, similar to the observed antibody responses, these data also suggest a compartment-specific regulation of cytokine responses. The authors further discuss that some of these inflammatory cytokines might influence local mucosal antibody production, although the interferon response did not show any obvious correlations with the virus-specific antibody response.

Viral load was also increased in but again poorly correlated between plasma and nasopharyngeal samples. Interestingly, while the viral load in the plasma increased with a more severe disease, the nasopharyngeal viral load was largely independent of the clinical presentation. Applying multidimensional scaling (MDS), the authors observed a positive association of the viral load with systemic inflammation and virus-specific antibody levels but not with the antiviral interferon response. Viral loads were also closely correlated with spike-specific IgG and IgA responses in nasopharyngeal samples. However, the authors discuss, in the context of MDS analysis, that the viral load was not the main driver of spike-specific antibody responses. In contrast, the viral load did not correlate with the inflammatory or regulatory cytokine response in mucosal tissue. Importantly, there was even a strong inverse association of the viral load with IL-33, colony-stimulating factor 3, and IFN-γ production (Fig. 1), again indicating distinct tissue-specific regulatory mechanisms.

**Fig. 1:**
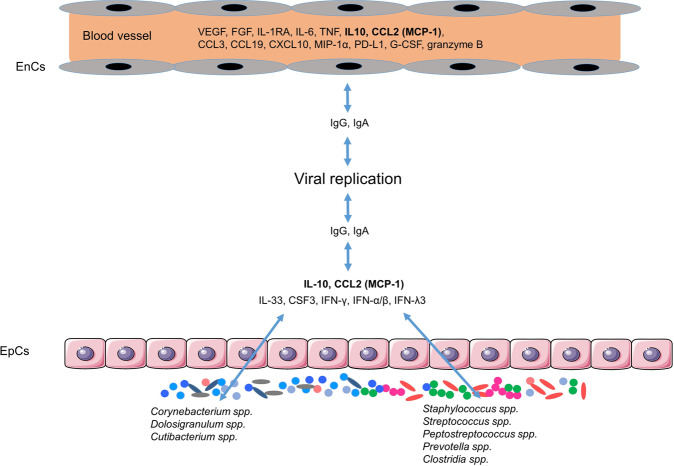
Compartmentalized immune responses characterize acute infection with SARS-CoV-2. Viral load correlates positively with humoral immune responses and the release of cytokines in the plasma. In contrast, SARS-CoV-2 infection either directly or indirectly disrupts the homeostasis of the local commensal microbiota in the nasopharynx, resulting in reduced levels of cytokines, including IL-33, IFN-γ, IFN-α/β, and IFN-λ3, that might be important for viral control. The accumulation of *Staphylococcus spp*. might thereby enhance viral load, whereas *Prevotella*, *Streptococcus*, *Peptostreptococcus*, and *Clostridia* drive the release of inflammatory cytokines and presumably aggravate disease independent of viral load. In contrast, *Corynebacterium* inhibit the expression of CCL2 and thus the influx of inflammatory monocytes into nasopharyngeal tissues. EnC endothelial cell, EpC, epithelial cell, VEGF vascular endothelial growth factor, FGF fibroblast growth factor, CSF-3 colony-stimulating factor 3

As a fourth variable, Smith et al. assessed the composition of the nasopharyngeal microbiota, which protects the epithelial surfaces of the host from colonization and/or invasion by different pathogens and pathobionts [9]. Thus, they performed a 16S rRNA sequence analysis of the microbiome in the nasopharynx of controls and patients with COVID-19 using V3–V4 region amplicons. Genus-level and alpha-diversity analyses revealed prominent perturbations in the composition of the microbiota from COVID-19 patients compared to that from healthy controls. The richness of the microbiota communities, as measured by β-diversity analysis, also significantly decreased with the severity of the disease. Specifically, the abundances of commensal *Corynebacterium* and *Dolosigranulum* were markedly reduced in COVID-19 patients in a severity-dependent fashion. Interestingly, IL-33, IFN-λ3, IFN-γ, or EGF was linked to microbial α-diversity and to the presence of *Corynebacterium* (Fig. 1), suggesting genus-specific and community-driven regulation of mucosal cytokine production. Interestingly, there was a negative correlation of microbial diversity and *Corynebacterium* with the chemokine CCL2 (Fig. 1), which recruits CCR2-expressing monocytes into infected and/or inflamed tissues [10]. In contrast, *Staphylococcus spp*. and several strict anaerobes, including the *Peptostreptococcus* and *Prevotella* genera, were enriched. The viral load in the nasopharynx correlated more closely with the abundance of *Staphylococcus*, whereas cytokines that were associated with severe disease were associated with an accumulation of other pathobionts, including *Prevotella*, *Streptococcus*, *Peptostreptococcus*, and *Clostridia* (Fig. 1). As the authors excluded smoking and sex as confounding factors and as nasopharyngeal swabs were collected before initiation of any antibiotic treatment, these results again suggest that SARS-CoV-2 infection might induce dysbiosis in nasopharyngeal microbial communities. However, as discussed by the authors, some specific bacterial communities might have already been present in the respective individuals before SARS-CoV-2 infection.

In summary, the data revealed unexpected relationships among the local mucosal microbiota and the systemic viral load, spike-specific antibody responses, and inflammatory cytokine levels. Although these data suggest inflammatory cytokines and local mucosal pathobionts as potential drivers of SARS-CoV-2 infection, further studies need to delineate in detail whether such nasopharyngeal dysbiosis perpetuates systemic inflammation, viral pathogenesis, clinical outcome, and/or viral transmission. Alternatively, alterations in the composition of the nasopharyngeal microbiota might precede SARS-CoV-2 infection and subsequently influence the clinical outcome of infection. Indeed, carriage of pathobionts such as *Staphylococcus aureus*, *Streptococcus pneumoniae*, or *Haemophilus influenzae* in up to 40% of healthy individuals [9] might predispose these individuals to severe COVID-19. Thus, SARS-CoV-2 infection might result in a disruption of local epithelial barrier function, leading to the escape of potential pathobionts and resulting in systemic manifestations. In summary, the study identifies new interactions among the virus, the microbiota, and the immune response of the host during infection with SARS-CoV-2, which may help to uncover new strategies for identifying individuals at risk for developing severe disease. However, the respective triggers of inflammatory cytokine production in hyperinflammatory COVID-19 syndrome need to be identified.

## References

[1] Smith N, Goncalves P, Charbit B, Grzelak L, Beretta M, Planchais C (2021). Distinct systemic and mucosal immune responses during acute SARS-CoV-2 infection. Nat Immunol.

[2] Williamson EJ, Walker AJ, Bhaskaran K, Bacon S, Bates C, Morton CE (2020). Factors associated with COVID-19-related death using OpenSAFELY. Nature.

[3] Li H, Liu L, Zhang D, Xu J, Dai H, Tang N (2020). SARS-CoV-2 and viral sepsis: observations and hypotheses. Lancet.

[4] Schultze JL, Aschenbrenner AC (2021). COVID-19 and the human innate immune system. Cell.

[5] Cervia C, Nilsson J, Zurbuchen Y, Valaperti A, Schreiner J, Wolfensberger A (2021). Systemic and mucosal antibody responses specific to SARS-CoV-2 during mild versus severe COVID-19. J Allergy Clin Immunol.

[6] Zhou R, To KK, Wong YC, Liu L, Zhou B, Li X (2020). Acute SARS-CoV-2 infection impairs dendritic cell and T cell responses. Immunity.

[7] Bastard P, Rosen LB, Zhang Q, Michailidis E, Hoffmann HH, Zhang Y, et al. Autoantibodies against type I IFNs in patients with life-threatening COVID-19. Science. 2020;370:eabd4585. 10.1126/science.abd4585. 10.1126/science.abd4585 PMC7857397 32972996

[8] Hadjadj J, Yatim N, Barnabei L, Corneau A, Boussier J, Smith N (2020). Impaired type I interferon activity and inflammatory responses in severe COVID-19 patients. Science.

[9] Brugger SD, Bomar L, Lemon KP (2016). Commensal-pathogen interactions along the human nasal passages. PLoS Pathog.

[10] Shi C, Pamer EG (2011). Monocyte recruitment during infection and inflammation. Nat Rev Immunol.

